# Ceramic Material Processing Towards Future Space Habitat: Electric Current-Assisted Sintering of Lunar Regolith Simulant

**DOI:** 10.3390/ma13184128

**Published:** 2020-09-17

**Authors:** Xin Li Phuah, Han Wang, Bruce Zhang, Jaehun Cho, Xinghang Zhang, Haiyan Wang

**Affiliations:** 1 School of Materials Engineering, Purdue University, West Lafayette, IN 47907, USA; xphuah@purdue.edu (X.L.P.); wang3281@purdue.edu (H.W.); cho299@purdue.edu (J.C.); xzhang98@purdue.edu (X.Z.); 2 School of Electrical and Computer Engineering, Purdue University, West Lafayette, IN 47907, USA; zhan2748@purdue.edu

**Keywords:** electric current-assisted sintering, lunar regolith simulant, microstructure, properties

## Abstract

*In situ* utilization of available resources in space is necessary for future space habitation. However, direct sintering of the lunar regolith on the Moon as structural and functional components is considered to be challenging due to the sintering conditions. To address this issue, we demonstrate the use of electric current-assisted sintering (ECAS) as a single-step method of compacting and densifying lunar regolith simulant JSC-1A. The sintering temperature and pressure required to achieve a relative density of 97% and microhardness of 6 GPa are 700 °C and 50 MPa, which are significantly lower than for the conventional sintering technique. The sintered samples also demonstrated ferroelectric and ferromagnetic behavior at room temperature. This study presents the feasibility of using ECAS to sinter lunar regolith for future space resource utilization and habitation.

## 1. Introduction

The last human exploration on the Moon was nearly 50 years ago and significant efforts are currently on-going to explore the potential of future human habitation on the Moon. Challenges remain in several aspects including the extreme cost to travel to the Moon, the short travel span, and very limited on-site resources available for human habitation. For future long-term human exploration on the Moon, it is thus necessary to establish a sustained long-term lunar habitat [1,2,3] via effective utilization of the resources available on the Moon.

The main resources available on the Moon are the lunar regolith, which is abundant and mainly comprises silicate minerals. The silicates can be extracted to produce necessary items for habitation, such as lunar concrete [4,5,6] and Si-based devices [7]. To study the possibility of processing lunar regolith, simulants with similar compositions have been used to demonstrate the feasibilities of material processing on the Moon. Previous studies have demonstrated the processing of lunar simulant powders via various bulk ceramic sintering techniques, including conventional sintering [8,9], microwave sintering [10,11,12], solar sintering [13,14], 3D printing [15,16,17,18,19,20,21], direct laser fabrication [22], selective laser melting [23,24], and glass-forming techniques [25].

Despite the successful demonstrations of the resource utilization of lunar simulants, the extreme environments on the Moon hinder the practical utilization of these bulk ceramic processing methods. For example, the Moon has reduced gravity, which is about a sixth of the terrestrial gravity. This leads to difficulty in forming compacts and distortions are likely to happen during the sintering process [26,27,28]. At high temperatures and in reducing atmospheres, rapid evaporation of selected elements and/or compounds will lead to the formation of macropores [29]. 

To possibly overcome the challenges of processing on the Moon, electric current-assisted sintering (ECAS), also known as spark plasma sintering, could be a viable technique for ceramic sintering under reduced temperatures and reduced gravity. ECAS is a sintering technique where powder is placed in an enclosed graphite die with applied pressure and high currents resistively heating up the die [30]. This technique offers high heating rates and applied pressure, which allow for rapid densification of ceramics to occur and result in highly dense samples in a very short time. 

In this work, we have demonstrated the utilization of ECAS for the sintering of lunar soil simulant JSC-1A with two different temperatures and pressures (550 °C, 30 MPa and 700 °C, 50 MPa). Various mechanical and physical properties, including mechanical hardness and ferromagnetic and ferroelectric properties, were analyzed to explore the great potential of lunar resource utilization of functional bulk ceramic materials for ferromagnets, ferroelectrics, and structural components on the Moon.

## 2. Materials and Methods

The lunar regolith simulant (JSC-1A) was sourced from Johnson Space Center. Table 1 shows the constituents in the powder, as previously reported [31]. The powder was placed in a plastic jar containing 1 mm diameter yttria-stabilized zirconia balls and was dry milled for 24 h in a horizontal ball mill to reduce the particle size. The powder was calcined in an alumina crucible at up to 700 °C for 4 h to remove moisture and possible volatile compounds. Approximately 0.8 g of powder was sintered in a 10 mm graphite die in a spark plasma sintering system (SPS, Thermal Technologies LLC, Santa Rosa, CA, USA). Two sintering conditions were compared: (1) 550 °C with an applied pressure of 30 MPa and (2) 700 °C with a pressure of 50 MPa. Both samples were heated at a rate of 100 °C/min and a pressure ramping rate of 10 MPa/min in a low-vacuum atmosphere (~10^−3^ torr). The system was cooled immediately (~200 °C/min) after reaching the maximum temperature. 

The powder and sintered samples were analyzed by X-ray diffraction (XRD, PANalytical Empyrean, Westborough, MA, USA) using Cu-kα radiation (λ = 0.154 nm). The powders and fracture surfaces were imaged by scanning electron microscope (SEM, Thermo Fisher NovaNanoSEM, Hillsboro, OR, USA) using an accelerating voltage of 10 kV. The powders and fracture surfaces were sputter-coated with Pt prior to imaging to prevent charging. The particle size distribution was measured from several micrographs using ImageJ (v1.49). Transmission electron microscopy (TEM) was performed on TALOS F200X TEM/STEM with ChemiSTEM technology (X-FEG and SuperX EDS with four silicon drift detectors, Hillsboro, OR, USA) at 200 kV for microstructure characterization and elemental mapping. For TEM observations, the specimen was mechanically ground and dimpled, followed by ion polishing in a precision ion milling system (PIPS II, Gatan, Pleasanton, CA, USA) for electron transparency.

The density of the sintered sample was measured by volumetric measurements using calipers and Archimedes density measurement by immersion in water. The density obtained from Archimedes measurements was compared to the density calculated from volume measurements with a caliper to ensure the density was consistent. The relative density (%) was obtained by calculating the ratio of measured density to the theoretical density of 2.9 g/cm^3^ and multiplying it by 100.

Prior to conducting mechanical testing, the specimen was polished by a series of fine diamond papers. A microhardness tester (LM 247AT, LECO Corporation, St. Joseph, MI, USA) equipped with a Vickers tip was employed for microhardness measurements. Twenty indents were made with a load of 200 gf and lengths of the diagonal indentations were measured under an optical microscope. For ferroelectric testing, gold contacts were deposited by pulsed laser deposition and polarization electric field (P-E) measurements were performed using a Precision LC II Ferroelectric Tester (Radiant Technologies Inc, Alburquerque, NM, USA) at room temperature. Magnetization properties were measured in a magnetic property measuring system (MPMS, Quantum Design, San Diego, CA, USA) at room temperature.

## 3. Results and Discussion

**Figure 1:**
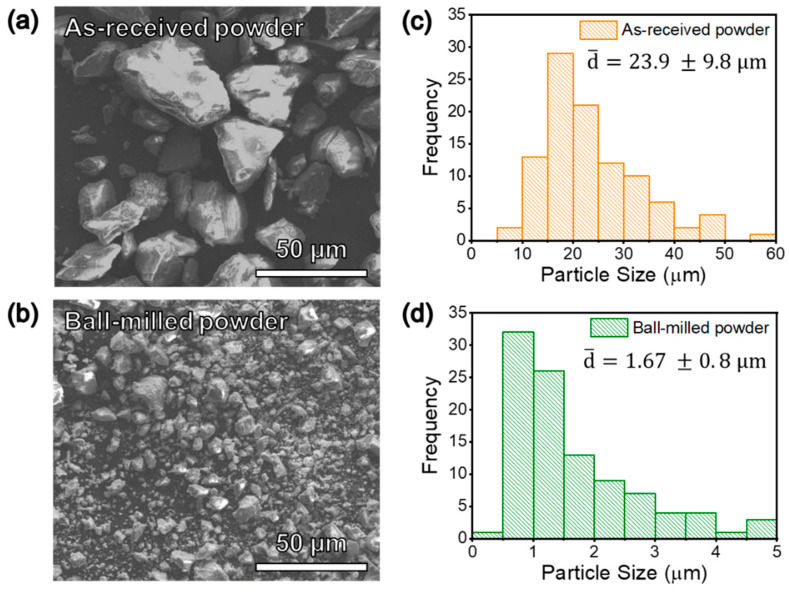
SEM images of the (**a**) as-received and (**b**) ball-milled JSC-1A powder. The corresponding particle size distributions are shown in (**c**,**d**).

Figure 1a,b shows the SEM images of the as-received and ball-milled JSC-1A powders at the same magnification. The as-received powder has mostly angular and sub-rounded particles, with a wide range of particle sizes [25]. There is a very clear reduction in average particle size from 23.9 μm to 1.67 μm after ball milling, which is shown by the particle size distributions in Figure 1c,d. The ball milling step can significantly reduce and homogenize the particle sizes for improved densification.

**Figure 2:**
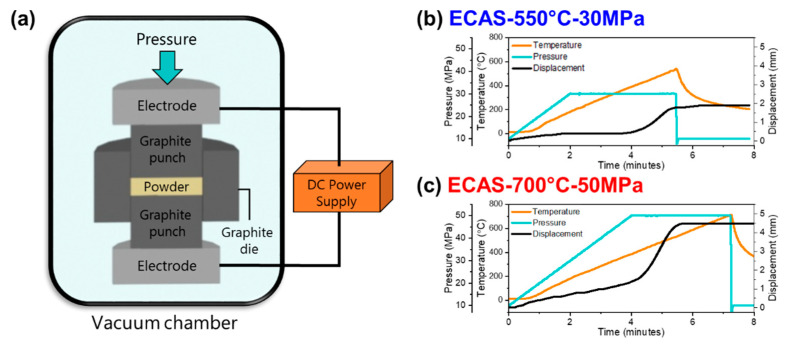
(**a**) Schematic of a spark plasma sintering system. Temperature, pressure, and displacement plots for the sintered JSC-1A samples: (**b**) ECAS-550 °C-30 MPa and (**c**) ECAS-700 °C-50 MPa.

Figure 2a shows the schematic of the ECAS system utilized to sinter the lunar regolith simulant. This sintering technique minimizes the tooling required, as the powder is contained, pressed, and sintered within the same tool. The two main parameters in ECAS which contribute significantly to the sintering process are temperature and pressure. Since pressure also provides an additional driving force for densification, the sintering temperature required will typically be lower than for conventional sintering. Additionally, the entire process is performed in vacuum atmosphere, which more closely resembles the Moon’s atmosphere. 

As such high temperatures and pressures may be challenging to achieve, exploring the effects of utilizing a lower temperature and pressure on the microstructure and bulk properties is necessary. The sintering temperatures were selected based on the displacement plots in Figure 2b,c at 30 and 50 MPa, respectively. Both samples showed changes in displacement due to sample shrinkage from densification and once the displacement began to plateau, the heating was stopped and immediately cooled. The lower pressure had less densification when plateaued at a lower temperature (550 °C), while the sample with higher pressure had greater shrinkage and heated to a higher temperature (700 °C). 

Both samples utilized lower temperatures than previous studies processed by conventional sintering, which are usually in the range of 1050–1200 °C [8,9,15]. The reduced particle size and applied pressure during sintering are the main contributions to the reduction of sintering temperature. Additionally, the rapid heating rate allows the entire process to only take a few minutes to complete, which is significantly shorter than conventional sintering. This also prevents volatile compounds from being evaporated, which form macropores in the sample [29]. 

**Figure 3:**
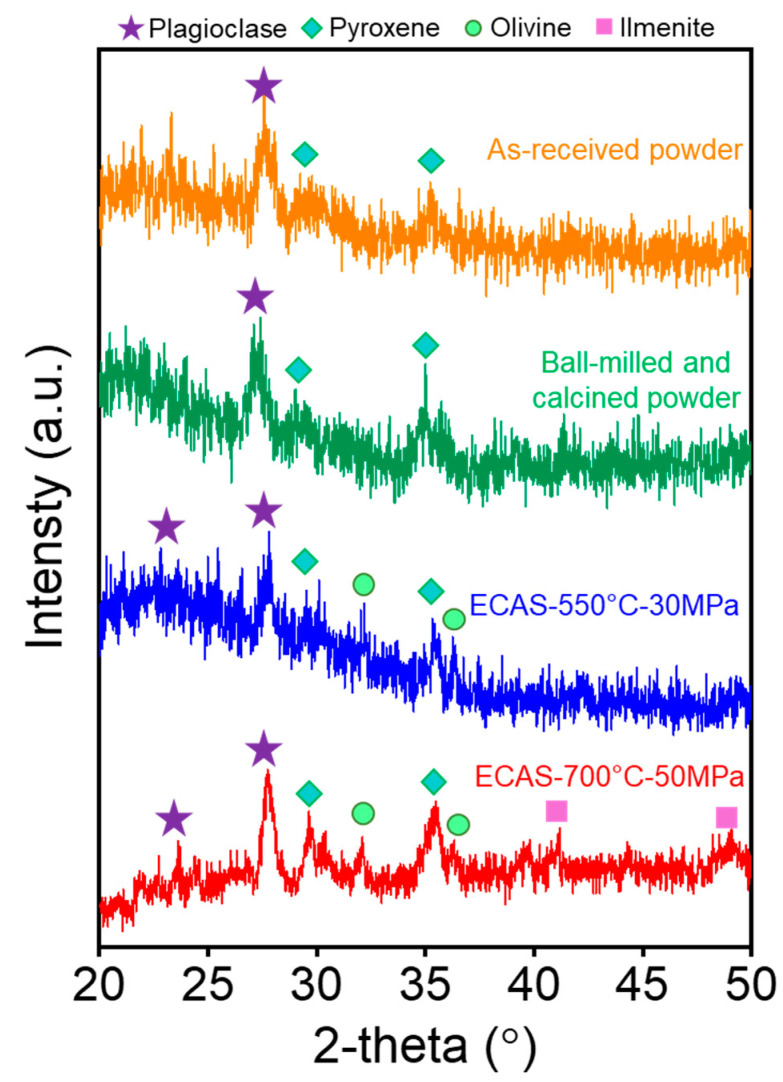
XRD pattern for the powder and sintered JSC-1A samples.

Figure 3 is the XRD spectra obtained from the as-received lunar simulant, processed powder, and the two sintered samples. The major phase present in the lunar regolith is plagioclase, which is a feldspar group, ranging from anorthite (CaAl_2_Si_2_O_8_) to albite (NaAlSi_3_O_8_) depending on the composition of Ca and Na. The other minerals in minor proportions include pyroxene (Ca,Mg,Fe)(Si, Al)_2_O_6_, olivine ((Mg,Fe)_2_SiO_4_), and ilmenite (FeTiO_3_). There was no obvious difference in phases between the as-received powder and the powder after ball milling and the calcination step. The ECAS-550 °C-30 MPa sample had very similar phases to the lunar simulant powders, where a broadened amorphous peak near low 2-theta angles (20–30°) was observed. By simply increasing the sintering temperature and pressure to 700 °C and 50 MPa, the crystallinity was increased as the amorphous broadening was removed. Although the JSC-1A powder has a glass crystallization temperature (T_c_) of 880 °C, which is higher than the sintering temperature [25], the combination of sintering temperature and pressure is likely to be sufficient for the sample to undergo recrystallization.

**Figure 4:**
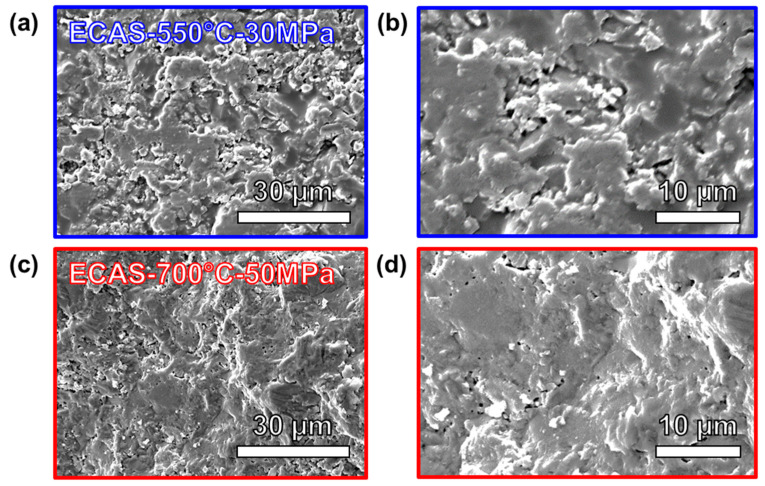
Fracture surface of (**a**,**b**) ECAS-550 °C-30 MPa and (**c**,**d**) ECAS-700 °C-50 MPa.

Figure 4 shows the fracture surface of the two sintered samples with different sintering conditions. Overall, the ECAS-700 °C-50 MPa sample was nearly fully dense, and had smaller pores compared to ECAS-550 °C-30 MPa. This is in agreement with the total displacement during the sintering process. With a higher sintering temperature and external applied pressure, the sample had a higher driving force for densification to occur and this removed more porosity.

**Figure 5:**
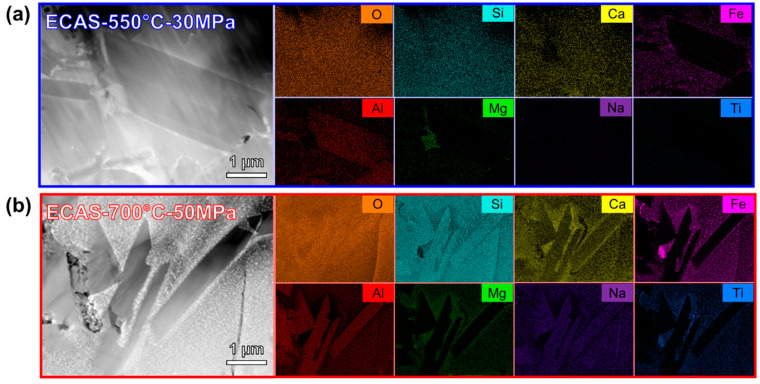
Scanning transmission electron microscopy (STEM) high-angle annular dark field (HAADF) images of (**a**) ECAS-550 °C-30 MPa and (**b**) ECAS-700 °C-50 MPa with the elemental mappings of elongated grains observed in the microstructure.

Figure 5 shows the scanning transmission electron micrograph (STEM) high-angle annular dark field (HAADF), along with the elemental mapping results. Elongated grains were found in the microstructure for both samples among the glassy phases. The elongated grains found in ECAS-550 °C-30 MPa contained O, Si, Ca, and Al elements, while ECAS-700 °C-50 MPa contained O, Si, Ca, Al, and Na. The elements contained within the elongated grains correspond to plagioclase. The remaining matrix for both samples contains Fe, Mg, Ca, and Ti, which likely corresponds to pyroxene.

Table 2 shows the relative density and microhardness of the ECAS-550 °C-30 MPa and ECAS-700 °C-50 MPa samples. As there was less total displacement observed for the ECAS-550 °C-30 MPa sample, the resulting density is slightly lower than in the ECAS-700 °C-50 MPa. This agrees well with the amount of porosity observed in the SEM images in Figure 4a,b. The microhardness values of ECAS-550 °C-30 MPa and ECAS-700 °C-50 MPa were measured to be 5.49 and 6.01 GPa, respectively. These microhardness values are comparable to glass materials, such as commercial and additive manufactured soda-lime glass [32,33]. This can be further improved by reaching full density with higher sintering temperatures, higher sintering pressures, and/or longer sintering times.

**Figure 6:**
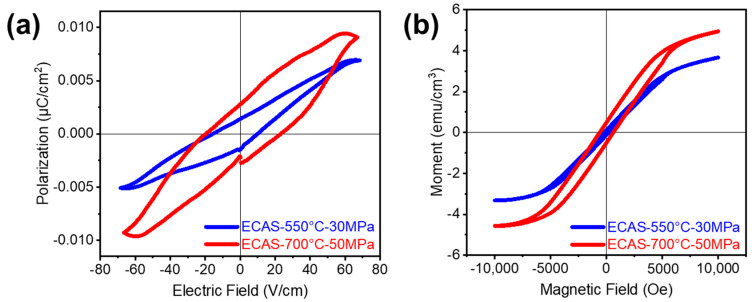
Room temperature (**a**) polarization electric field (P-E) and (**b**) magnetization magnetic field (M-H) hysteresis loops of ECAS-550 °C-30 MPa and ECAS-700 °C-50 MPa.

Since the lunar regolith contains mostly dielectric oxides and a substantial amount of Fe species (~10 wt %), ferroelectric and magnetic behavior could be expected. The polarization electric field (P-E) hysteresis loops are clearly shown for both sintered samples in Figure 6a, where the ECAS-700 °C-50 MPa sample demonstrated a higher saturated polarization compared to ECAS-550 °C-30 MPa. The magnetization hysteresis loops (M-H loops) of the two sintered samples were compared at room temperature in Figure 6b. The different sintering conditions led to contrasting magnetic behavior, and ECAS-700 °C-50 MPa demonstrated a higher saturation and larger coercivity than those of the ECAS-550 °C-30 MPa sample. This shows that both the ferroelectric and ferromagnetic properties are stronger when sintered at a higher temperature and pressure. The major reason for the improvement in both properties could be attributed to enhanced densification. As ECAS-550 °C-30 MPa has a lower density, more porosity would be detrimental to the dielectric breakdown susceptibility [34] and decrease the magnetic permeability due to the interparticle gap effect [35].

The multiferroicity (both ferroelectric and ferromagnetic, for this case) in the sintered lunar regolith came from the following properties. The mineral containing Fe is pyroxene, which is a minor phase in the lunar regolith. Pyroxene was recently reported as a class of multiferroic material in 2007 [36]. Only several pyroxenes have been previously investigated, including NaFeSi_2_O_6_ [37,38], which could be the main contributor to the ferroelectric and ferromagnetic behavior of the lunar regolith based on the constituents of the lunar simulant. These promising functionalities suggest that sintered lunar regolith could be used as not only structural ceramic, but also a functional ceramic for sensors, actuators, magnetometers, and antennas using *in situ* resources on the Moon for sustainable human habitation.

## 4. Conclusions

*In situ* resource utilization is critical for future space habitation on the Moon. The use of ECAS to consolidate the lunar regolith was investigated as a potential method of material processing on the Moon. Using ECAS can significantly reduce the sintering temperature and time for a more efficient processing rate. At 700 °C and 50 MPa, the final density reached 97% and the microhardness was similar to other glass materials. The lunar regolith simulant also exhibited tunable ferroelectric and magnetic behavior, where higher polarization and magnetization can be achieved with higher sintering temperature and pressure. Since the differences in properties between the two sets of conditions in this study are rather minimal, using the lower temperature and pressure sintering conditions could be sufficient for some of the applications, such as building blocks for human habitats. ECAS would not only provide a single-step solution to densifying compacts, but could also help to overcome the challenges of sintering in an extreme atmosphere on the Moon. Modification of ECAS tooling to be suitable for deployment on the Moon or a space station could be an important step towards future long-term *in situ* resource utilization in space habitats. 

## Figures and Tables

**Table 1:** Chemical composition of the oxides in JSC-1A simulant [31].

Oxide	Concentration (wt %)
SiO_2_	47.10
TiO_2_	1.87
Al_2_O_3_	17.10
Fe_2_O_3_	3.41
FeO	7.57
MnO	0.18
MgO	6.90
CaO	10.30
Na_2_O	3.30
K_2_O	0.86
P_2_O_5_	0.76

**Table 2:** Measured density and microhardness of ECAS-550 °C-30 MPa and ECAS-700 °C-50 MPa.

Sample	Relative Density (%)	Hardness (GPa)
ECAS-550 °C-30 MPa	95	5.49 ± 0.53
ECAS-700 °C-50 MPa	97	6.01 ± 0.66
